# Variation in presenteeism by generosity of statutory sick pay: a multilevel analysis in 35 European countries

**DOI:** 10.1093/eurpub/ckag093

**Published:** 2026-06-12

**Authors:** Marvin Reuter, Tabea Gau, Sophie-Charlotte Meyer, Sascha A Ruhle

**Affiliations:** Junior Professorship for Sociology, esp. Work and Health, Department of Sociology, University of Bamberg, Bamberg, Germany; Junior Professorship for Sociology, esp. Work and Health, Department of Sociology, University of Bamberg, Bamberg, Germany; Division Changing World of Work, Federal Institute for Occupational Safety and Health, Dortmund, Germany; Department of Human Resource Studies, Tilburg School of Social and Behavioural Science, Tilburg University, Tilburg, The Netherlands

## Abstract

Attending work despite illness (sickness presenteeism) is common in many workplaces. While most research has focused on individual and workplace determinants, less is known about country-level factors. This study examines (i) the association between national sick pay policies and presenteeism, (ii) the extent to which these policies contribute to cross-country variation in presenteeism, and (iii) whether associations differ by sociodemographic and occupational characteristics. We used data on 19 657 employees in 35 countries from the 2015 European Working Conditions Survey combined with country-level information on sick pay regulations. Countries were classified as offering ‘generous’ sick pay if they compensated from the first day of illness with ≥80% wage replacement for at least 2 weeks (43% of the countries met this criterion). Presenteeism was measured by the fraction of days worked while ill out of all illness days in the past 12 months (presenteeism propensity). We estimated multilevel models that controlled for individual characteristics (sociodemographics, job characteristics, health) and country features (unemployment, gross domestic product, and population density). Generous sick pay was associated with an 8-percentage-point lower presenteeism propensity (average marginal effect: −0.08; SE: 0.04; *P *< .05), explaining 12.4% of the between-country variance in presenteeism propensity. The association appeared more pronounced among older workers, low-income employees, routine occupations, and those in industry or public administration. National sick pay regulations may shape presenteeism, particularly among groups where financial motives are central. Adequate sick pay may help prevent workers from engaging in presenteeism and mitigate its consequences.

## Introduction

Sickness presenteeism, originally described as the flipside of absenteeism [1], refers to the behaviour whereby workers attend work despite illness. In Europe, >40% of the workforce engages in presenteeism at least once a year [2], making it a common phenomenon in the workplace. Presenteeism can have detrimental effects on their own health and that of others, either by facilitating the spread of infectious diseases or impeding recovery, which may increase the risk of exacerbating or recurring health issues in the future [3, 4]. These health consequences can, in turn, also lead to productivity losses [5]. Given its various negative effects, understanding the factors that drive employees to engage in presenteeism is crucial for developing policies.

In the past, research has primarily focused on individual and workplace correlates of presenteeism, such as job demands and heavy workloads [6, 7], overtime work [8], poor leadership quality [6], work attitudes like a strong sense of duty [4] and organizational policies such as attendance monitoring and penalties for absences [6, 9]. Other contributing factors include financial difficulties [10] and job insecurity [11].

In addition to individual and organizational factors, country features on the macro-level may also play an important role. Within Europe, presenteeism has been found to vary notably between countries [11]. However, research on the broader contextual factors affecting the likelihood of presenteeism is sparse. It has been shown that workers in regions with higher unemployment rates are more likely to choose presenteeism, likely due to concerns about job security [12]. Societal norms in the perception of presenteeism as an accepted behaviour could also play a role. For example, China has higher rates of presenteeism than the UK, which can be partially explained by Confucian values of hard work and persistence [13].

A key macro-level factor may be the generosity of national sick pay schemes. The financial burden of sickness leave varies widely across countries, depending on the extent and timing of wage compensation. In social security systems with limited wage replacement, employees are more likely to face wage penalties when taking sickness absence. In contrast, countries with more generous sick pay schemes reduce financial disincentives, encouraging workers to prioritize recovery over presenteeism. Rostad *et al*. [14] have compared sick pay schemes of Sweden, Norway and Italy for physicians, a group that often attends work despite being ill . They discovered that in Italy, the country with the least generous sick pay provisions, physicians have higher scores of presenteeism than in Sweden and Norway respectively. In the United States, where no guaranteed federal sick pay exists, the introduction of local sick pay legislation in certain industries has been associated with reduced presenteeism [15, 16]. As data rarely include direct measures of presenteeism, many studies infer it from reductions in sickness absence rates. In Sweden, reforms that increased sick pay generosity were associated with a rise in sick leave days, while cuts to benefits were followed by a decline [17]. Similar trends have been observed in Germany [18, 19]. In contrast, Pollak [20] found that employees in France who received immediate compensation instead of a three-day waiting period took shorter and not more sick leaves.

While sick pay schemes are likely to play an important role in shaping workers’ presenteeism and explaining cross-country variation, existing research remains limited. Most studies to date have focused either on time-trend analyses within a single country or on cross-country comparisons involving only a small number of nations. Analysing data from a larger and more diverse set of countries would enable a more systematic assessment of contextual variation in presenteeism and the relative contribution of sick pay schemes, while accounting for other contextual factors. Second, many studies use absenteeism as an indirect proxy for presenteeism, based on the assumption that increases in sick leave reflect a reduction in presenteeism [19, 20]. However, this inference is speculative, as changes in absenteeism may also reflect underlying shifts in population health rather than altered work-rest decisions. Third, most analyses focus solely on wage replacement rates, overlooking other key elements of sick pay systems. A combined consideration of wage replacement, duration of coverage (how long compensation is provided), and the presence or absence of unpaid waiting periods (whether compensation begins immediately) would more comprehensively capture the financial risks of taking sick leave. Finally, few studies examine how individual circumstances moderate the relationship between sick pay and presenteeism. Workers with limited financial resources are likely to be more sensitive to compensation regulations, whereas for those in higher class positions or with more severe illnesses, financial incentives may play a smaller role in the decision to attend work while sick.

This study adopts a cross-country perspective, examining variation in presenteeism across 35 European nations and its association with state-level sick pay schemes. According to Moral Hazard Theory, workers in countries providing more generous sick pay are expected to engage less frequently in presenteeism because they do not to face wage penalties of absenteeism [21, 22]. Accordingly, the central research questions are whether presenteeism is lower in countries with more generous sick pay schemes and if sick pay explains cross-national variation in presenteeism. We further examine whether the association between sick pay and presenteeism varies by sociodemographic and occupational characteristics.

## Methods

### Data

We combined data on presenteeism collected in the 2015 European Working Conditions Survey (EWCS) with country-level information on national sick pay regulations. The EWCS is a cross-sectional trend survey designed to capture various aspects of working conditions and employment quality across European countries [23]. A strength is that it provides direct measures of workers’ presenteeism across a large number of countries using a common methodological approach. The 2015 wave was specifically selected because it captures workers’ sickness behaviours prior to being influenced by the heightened infection risks and the social and legal restrictions introduced during the COVID-19 pandemic. At the time of writing, the post-pandemic wave of the EWCS, scheduled for 2025, was not yet available. The 2015 EWCS consists of nationally representative samples drawn from 35 countries, comprising current and former European Union Member States as well as Norway, Switzerland, Albania, North Macedonia, Montenegro, Serbia, and Turkey. Participants were selected through multistage, stratified random sampling procedures within each country. Data were collected via face-to-face interviews conducted at respondents’ homes between February and September 2015, achieving an average response rate of 43%.

The EWCS data were enriched by integrating country-level information on national sick pay regulations using the Sick Pay Database [24]. This database provides harmonized indicators of sick pay policies in place on 1 July 2015, corresponding to the 2015 EWCS. Definitions and constructs were developed by the World Policy Analysis Center (WPAC) [25] and the comparative tables of the Mutual Information System on Social Protection (MISSOC) [26] and were provided in the form of categorical variables. For Greece, reliable 2015 information was unavailable, so we approximated Greek sick pay provisions using more current data, based on the observation that regulations have remained relatively stable over time. All data sources used in this study are publicly accessible, fully anonymized, and freely available for scientific research purposes. No additional ethical approval was required.

### Study sample

The initial EWCS sample included observations from 43 850 individuals. The analytical sample for this study was derived through a sequential process. First, we excluded 1979 respondents who were unemployed or economically inactive at the time of the survey. Second, 7866 interviews with self-employed individuals were excluded, since national sick pay policies primarily target employees. Third, we excluded 568 individuals who were younger than 18 years or older than 65 years to focus the analysis on individuals primarily depending on employment income. Fourth, we imputed missing data using chained equations, as non-response was high in the question on sickness absence days in the past 12 months (11.5%), probably reflecting recall difficulties (for details see Table S1). A listwise deletion would likely have led to biased estimates (MCAR test: *P* < .001) [27]. The distributions of variables after imputation closely matched the original data (Table S2). Fifth, in line with previous research [28], we excluded 387 participants who reported exceptionally high sickness absence or presenteeism days (exceeding 70 days per year), as financial considerations may not have primarily influenced presenteeism decisions within this subgroup. Sixth, we removed 13 393 respondents who reported zero days of sickness absence and presence in the last year, as these individuals did not experience any sickness episodes requiring a decision between absenteeism and presenteeism. The final analytical sample consists of 19 657 employees from 35 European countries.

### Variables

#### Presenteeism propensity

We combined participants’ self-reported number of sickness-related absence days with the number of days they reported working despite illness over the preceding 12 months. For each participant, we then calculated the proportion of presenteeism days relative to the total number of days with health issues. This measure, referred to as presenteeism propensity, offers a more advanced approach to examining individuals’ choices to engage in presenteeism, as it allows comparison of individuals with different health conditions or different numbers of working days [29]. Scores on the presenteeism propensity measure range from 0 (no days worked while ill) to 1 (all illness days spent working). As a robustness check, we analysed sickness absence days and presenteeism days as alternative outcome measures (see Sensitivity analyses).

#### Sick pay generosity

Although all European countries guarantee some form of monetary compensation during sickness absence, the timing, duration, and generosity of these provisions vary substantially. In line with previous studies [17, 19], we focused on the level of monetary compensation provided during periods of sickness absence (wage replacement rate). However, our approach also incorporated information on waiting periods (i.e. whether sick pay is provided from the first day of illness) and duration (i.e. how many weeks of well-compensated sick leave are available). Countries were classified as providing ‘generous’ sick pay if individuals received compensation from the first day of sickness absence, with at least 80% wage replacement sustained for a period of 2 weeks or more. The two-week threshold follows the approach used by the WPAC [25] and captures the majority of sickness episodes observed in our data. By dividing countries into two groups, we aimed to capture sufficient heterogeneity within both groups to control for other country characteristics. As a robustness check, we also examined whether the results differed when using single policy indicators or a composite sum score (see Sensitivity analyses).

#### Control variables

To adjust for variations in socio-structural composition and labour market characteristics across countries, including non-statutory regulations towards sick pay, we incorporated both individual-level and country-level covariates in our analyses (see conceptualization in Fig. S1). At the individual level, we controlled for sociodemographic factors, job characteristics, and health. Sociodemographic variables include age, sex, household composition, foreign-born status, education (International Standard Classification of Education, ISCED-2011), and difficulty making ends meet based on household income. Job characteristics comprised occupational class (European Socioeconomic Classification, ESeC), working sector (Statistical Classification of Economic Activities in the European Community, NACE), employment contract, working hours, job tenure, company size, and union or works council representation. Unionization is a strong predictor of company sick pay that can vary between countries and affect the presenteeism propensity [12]. Health indicators included self-rated health, physical health problems reported in the past 12 months (e.g. hearing issues, skin problems, backache, upper and lower muscular pain, headaches, injuries), long-standing illnesses (≥6 months), mental health (WHO-5 Well-Being Index), and health events (sum of sickness absence and presence days). At the country level, we considered the unemployment rate, gross domestic product (GDP) per capita, and population density (people per square kilometre). A high unemployment rate may indicate greater labour market insecurity; GDP per capita serves as a proxy for a country’s economic wealth; and population density reflects the degree of urbanization. Information on macro-level indicators corresponding to the EWCS survey year was obtained from Eurostat and the OECD [30, 31].

### Statistical analysis

We performed several analytical steps to investigate the relationship between sick pay schemes and presenteeism propensity. First, we described countries with and without generous sick pay schemes by means and frequencies of the dependent and control variables. Second, we performed a series of multilevel regression models to investigate the association between sick pay generosity and presenteeism propensity [32]. Multilevel models account for the hierarchical data structure where observations (individuals) are nested within groups (countries), allowing more accurate estimation of standard errors and the decomposition of variance in presenteeism propensity at both the individual- and country-level. As the presenteeism propensity variable was skewed due to a high frequency of the values ‘0’ (no days worked while sick) and ‘1’ (all days worked while sick), we employed a multilevel generalized linear model (MEGLM) with a binomial distribution and a logit link function [33]. To address the non-normality of the data, we used robust (sandwich) standard error estimation.

Our analytical strategy involved estimating a series of random intercept models. We began with an empty model without any covariates (Model 0) to quantify the variance in presenteeism propensity attributable to individual and country differences by calculating the intraclass correlation (ICC). We then sequentially added individual-level control variables (Model 1) and country-level control variables (Model 2), each to adjust for structural differences between countries. Finally, we introduced the sick pay generosity variable to estimate the association with presenteeism propensity (Model 3). In each model, we report variances at both the individual and country-level. As variances in logit models are not comparable across different models, we applied the rescaling method of McKelvey and Zavoina as described in Hox [32]. We interpreted a negative and statistically significant effect estimate of the sick pay variable in combination with a reduction of country-level variance as a confirmation of the assumption that more generous sick pay is associated with a lower tendency to choose presenteeism. In addition, we calculate a series of models that each include a cross-level interaction term between country sick pay and sociodemographic factors (sex, age, financial strains), job characteristics (occupational class, economic sector) and health events. To enhance the interpretability of the logit model results, we calculated average marginal effects (AMEs) [34]. These represent the average change in presenteeism propensity, expressed in percentage points, for a one-unit change in the predictor variable. Results from the interaction models were further expressed as predicted margins, comparing presenteeism propensities between generous and non-generous sick pay regimes across different social strata (e.g. by sex or age group). As part of sensitivity analyses, we estimated additional models using alternative sick pay and sickness behaviour indicators to assess the robustness of our findings. All analyses were performed using Stata 18 MP (64-bit, StataCorp LLC, College Station, TX, USA).

## Results

### Country differences

**Figure 1.**
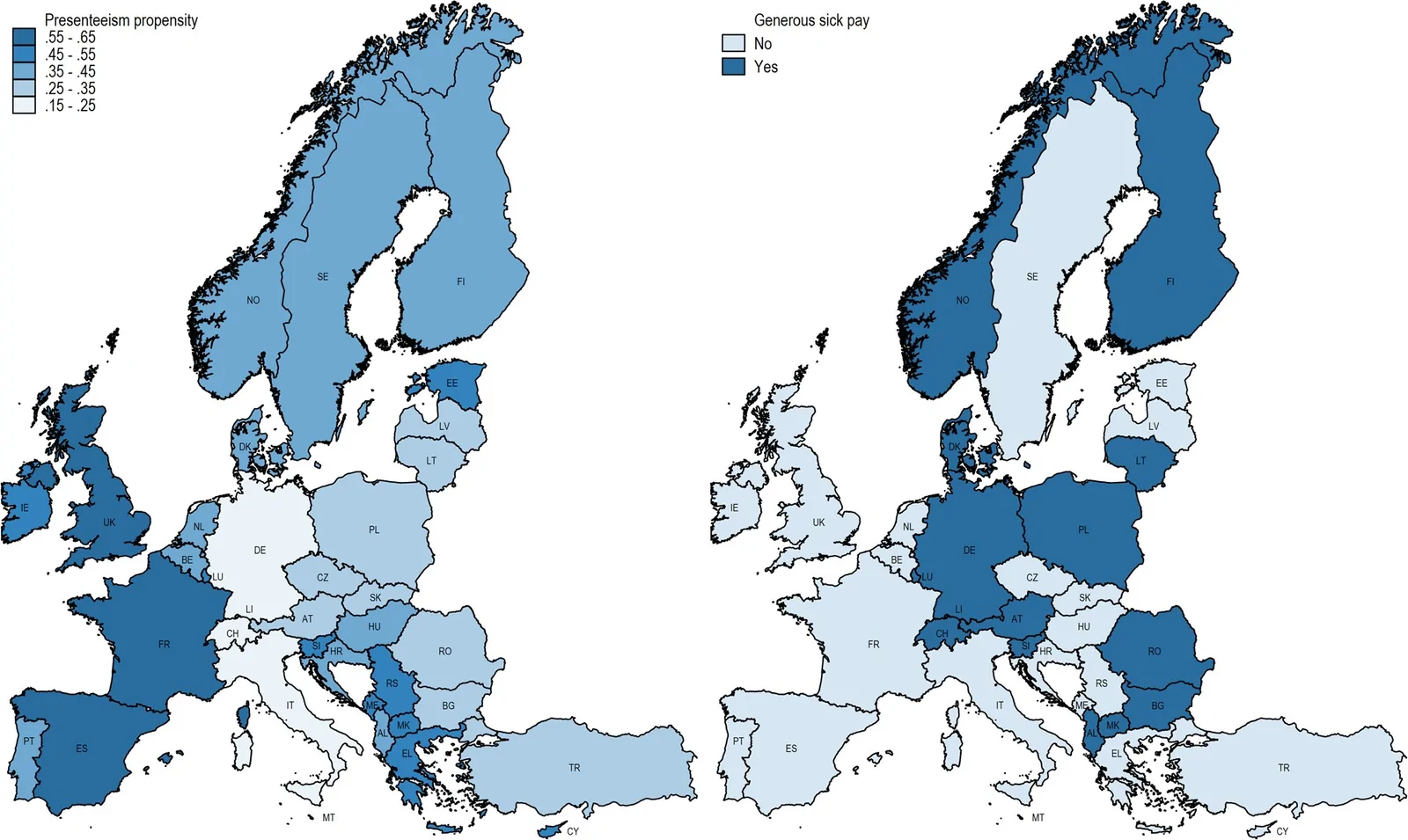
Mean presenteeism propensity (left) and classification of countries into generous and non-generous sick pay regimes (right). Generous sick pay was defined as compensation from the first day of sickness, with wage replacement at 80% for at least 2 weeks. Country abbreviations: AL, Albania; AT, Austria; BE, Belgium; BG, Bulgaria; CH, Switzerland; CY, Cyprus; CZ, Czechia; DE, Germany; DK, Denmark; EE, Estonia; EL, Greece; ES, Spain; FI, Finland; FR, France; HR, Croatia; HU, Hungary; IE, Ireland; IS, Iceland; IT, Italy; LT, Lithuania; LU, Luxembourg; LV, Latvia; ME, Montenegro; MK, North Macedonia; MT, Malta; NL, Netherlands; NO, Norway; PL, Poland; PT, Portugal; RO, Romania; RS, Serbia; SE, Sweden; SI, Slovenia; SK, Slovakia; TR, Türkiye; UA, Ukraine; UK, United Kingdom. A more detailed tabular representation is to find in the Supplementary file (Table S3).

The mean presenteeism propensity across all countries was 0.41 (SD 0.42), and 15 of the 35 countries (43%) were classified as having generous sick pay (Table S3). As shown in Fig. 1, presenteeism propensity varied substantially, ranging from 0.18 in Italy to 0.60 in France. A pattern emerged in which several Western European countries combined relatively high presenteeism propensities with non-generous sick pay, while many Central and some Eastern European countries had lower presenteeism propensities alongside generous sick pay. Northern and Southern Europe displayed a more heterogeneous picture.

### Sample description

The descriptive comparison between countries with and without generous sick pay in Table 1 reveals notable differences. Workers in countries with more generous sick pay exhibited on average higher levels of sickness absence, lower levels of sickness presence, and thus a lower presenteeism propensity. Countries with generous sick pay also tended to have a workforce that was older, better educated, more often native-born, and less financially strained. Occupationally, they featured more permanent contracts, a higher share of managers and industry jobs, and greater union or works council representation. Despite reporting more long-standing illnesses and poorer general health, workers in these countries had better mental health on average. Countries providing generous sick pay had lower unemployment rates, higher GDP per capita, and greater population density.

**Table 1.** Descriptive statistics.

		Non-generous sick pay		Generous sick pay		
Variables	Category or (range)	*N* (Col%)	Mean (SD)	*N* (Col%)	Mean (SD)	***P*** ^a^
**Sickness behaviour**						
Sickness absence	(0–70 days)		7.30 (11.77)		8.78 (12.07)	<.001
Sickness presence	(0–70 days)		4.62 (7.88)		4.31 (7.63)	.005
Health events	(0–130 days)		11.93 (14.72)		13.09 (14.91)	<.001
Presenteeism propensity	(0–1)		0.45 (0.42)		0.36 (0.40)	<.001
**Sociodemographic factors**						
Age	(18–65)		42.03 (11.25)		42.65 (11.31)	<.001
Sex	Male	5459 (46.6)		3609 (45.4)		.114
	Female	6257 (53.4)		4332 (54.6)		
Type of household	Single, no children	1779 (15.2)		1188 (15.0)		<.001
	Couple, no children	4001 (34.1)		2852 (35.9)		
	Couple with children	3588 (30.6)		2543 (32.0)		
	Single with children	662 (5.7)		345 (4.3)		
	Others	1,686 (14.4)		1013 (12.8)		
Foreign born	No	11 050 (94.3)		7584 (95.5)		<.001
	Yes	666 (5.7)		357 (4.5)		
Education	Primary and lower secondary	1985 (16.9)		972 (12.2)		<.001
	Upper secondary	5381 (45.9)		4055 (51.1)		
	Tertiary	4350 (37.1)		2914 (36.7)		
Making ends meet	Easily	6493 (55.4)		5154 (64.9)		<.001
	Some difficulties	4609 (39.3)		2514 (31.7)		
	Great difficulties	614 (5.2)		273 (3.4)		
**Job characteristics**						
Occupational class	Higher managers/professionals	1354 (11.6)		1145 (14.4)		<.001
	Lower managers/professionals	3067 (26.2)		2216 (27.9)		
	Lower supervisors, technicians	1756 (15.0)		1015 (12.8)		
	Lower sales and service	2506 (21.4)		1508 (19.0)		
	Lower technical	1509 (12.9)		1177 (14.8)		
	Routine	1524 (13.0)		880 (11.1)		
Working sector	Agriculture	202 (1.7)		92 (1.2)		<.001
	Industry	1879 (16.0)		1437 (18.1)		
	Construction	636 (5.4)		514 (6.5)		
	Transport	704 (6.0)		447 (5.6)		
	Commerce and hospitality	2201 (18.8)		1355 (17.1)		
	Financial services	459 (3.9)		349 (4.4)		
	Public administration	905 (7.7)		491 (6.2)		
	Education	1277 (10.9)		850 (10.7)		
	Health	1423 (12.1)		1043 (13.1)		
	Other services	2030 (17.3)		1363 (17.2)		
Type of working contract	Permanent	9372 (80.0)		6766 (85.2)		<.001
	Temporary	1390 (11.9)		732 (9.2)		
	Other	954 (8.1)		443 (5.6)		
Weekly working hours	(1–80)		38.06 (10.71)		38.00 (9.85)	.678
Job tenure (years)	(0–50)		10.04 (9.42)		10.40 (9.66)	.010
Company size	< 10	2444 (20.9)		1470 (18.5)		<.001
	10–249	4943 (42.2)		3540 (44.6)		
	250+	4329 (36.9)		2931 (36.9)		
Union or works council	No	5703 (48.7)		3535 (44.5)		<.001
	Yes	6013 (51.3)		4406 (55.5)		
**Health conditions**						
Self-rated health	(1 = very bad to 5 = very good)		3.96 (0.75)		3.92 (0.75)	<.001
Physical health problems	(0–7)		2.10 (1.61)		2.14 (1.58)	.059
WHO-5 well-being index	(0 = worst to 25 = best)		16.19 (5.29)		16.60 (4.91)	<.001
Long-standing illness	No	9211 (78.6)		6045 (76.1)		<.001
	Yes	2505 (21.4)		1896 (23.9)		
**Country-level factors**						
Unemployment rate (%)	(3.8–28.0)		12.66 (6.12)		8.12 (4.81)	<.001
GDP per capita (EUR/1000)	(3.5–92.8)		25.11 (12.16)		36.41 (26.13)	<.001
Population density	(16.9–1375.2)		157.64 (127.69)		232.38 (363.96)	<.001
**Total**		11 716 (59.6)		7941 (40.4)		

a Significance of group differences by Chi-square test (for categorical variables) or Pearson’s *r* (for continuous variables).

### National sick pay and presenteeism propensity

Table 2 shows the results of the multilevel regression analysis. In the empty model (Model 0), the ICC coefficient was 0.095, indicating that ∼9.5% of the variation in presenteeism propensity was due to country differences, whereas the remaining 90.5% was between individuals. Adding individual-level variables in Model 1 (full results in Table S4) revealed that the presenteeism propensity was higher among young workers, women, those facing financial strains, in higher class positions, in temporary jobs. A high presenteeism propensity was also linked to long working hours, a lack of union representation, and poorer health. These factors explained 17.9% of the individual-level variance and 15.1% of the country-level variance. Model 2 included country-level controls, showing that higher unemployment, GDP, and population density were linked to greater presenteeism propensity, reducing country-level variance by 32.2%. Model 3 introduced sick pay generosity, which was significantly associated with an 8–percentage–point lower presenteeism propensity. This final model further reduced between-country variance by 12.4%.

**Table 2.** Multilevel generalized linear regression models for presenteeism propensity.

	Model 0		Model 1		Model 2		Model 3	
	AME	(SE)	AME	(SE)	AME	(SE)	AME	(SE)
**Level 1 variables**			✓		✓		✓	
**Level 2 variables**								
**Unemployment rate (Std.)**					0.05***	(0.02)	0.05***	(0.01)
**GDP per capita (Std.)**					0.07***	(0.02)	0.08***	(0.02)
**Population density (Std.)**					0.04***	(0.01)	0.04***	(0.01)
**Generous sick pay**								
No							Ref.	
Yes							−0.08*	(0.04)
**Intercept**	0.57***	(0.02)	0.57***	(0.02)	0.59***	(0.02)	0.59***	0.0
**Variance component**								
**Level 1 (Individuals)**	3.290		2.700		2.700		2.700	
**Level 2 (Countries)**	0.345		0.293		0.199		0.174	
Intraclass correlation	0.095							
**Variance reduction**								
Level 1			−17.9%		0.0%		0.0%	
Level 2			−15.1%		−32.2%		−12.4%	
**Model information**								
***N* (Individuals)**	19 657		19 657		19 657		19 657	
***N* (Countries)**	35		35		35		35	

The full table including estimates for level 1 variables is to find in the Supplementary data (Table S4). AME, Average marginal effect (this represents the average change in the predicted fraction of days worked while sick, expressed in percentage points; e.g. on average, one additional unit in GDP was linked to a 7–percentage‐point higher fraction of presenteeism days in Model 2). SE, Standard error. Continuous variables have been standardized (Std.). Variances have been rescaled following the McKelvey and Zavoina method (as described in Hox 2010, 133–9) to make them comparable across different logit models.

* *P* < .05,

** *P* < .01,

*** *P* < .001.

### Subgroup analyses

**Figure 2.**
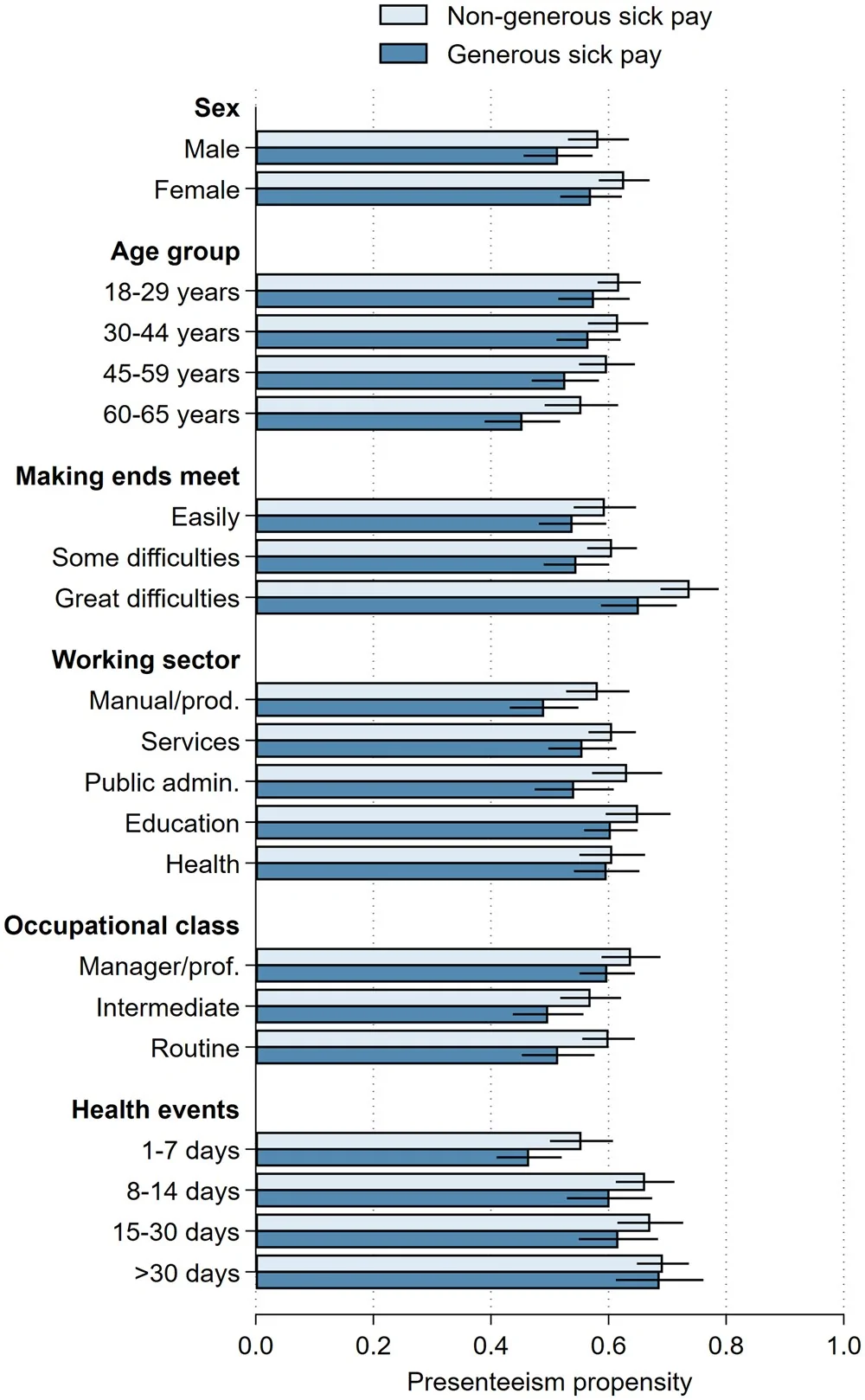
Predicted presenteeism propensity with 95% confidence intervals across workforce subgroups by sick pay generosity. Bars represent predicted margins obtained from a series of multilevel generalized linear regression models (19 657 individuals within 35 countries). Analyses controlled for household, foreign born, weekly working hours, job tenure, company size, self-rated health, physical health problems, long-standing illness, WHO-5 well-being index and on the country-level: unemployment rate, GDP and population density. Results in tabular form are presented in the Supplementary file (Table S5).

Figure 2 shows that most social groups had lower presenteeism propensities when living in countries providing generous sick pay. Based on predicted margins from interaction models, these patterns reflect adjusted, model-based differences. The gap appeared more pronounced among men, older workers, those reporting financial difficulties, employees in routine or intermediate occupational classes, and those working in production or public administration. By contrast, differences were small or absent among workers in managerial positions, those employed in the health care sector, and employees who experienced a high number of health events.

### Sensitivity analyses

To test the robustness of our findings, we estimated additional models using alternative operationalizations of sick pay generosity indicators (Table S6) and alternative outcome measures of sickness behaviour (Table S7). While neither the waiting period nor wage replacement rate in week 2 showed significant associations with presenteeism propensity when analysed alone, the absence of a qualifying period was linked to lower presenteeism propensity. A combined score sum incorporating all three dimensions (qualifying period, waiting period, and wage replacement) showed that each additional point in sick pay generosity was associated with reduced presenteeism propensity, with significant differences between the most and least generous countries. Moreover, analyses using alternative outcome measures (Table S7) indicated that more generous sick pay was related to more absenteeism days (*P* < .05) and fewer presenteeism days (n.s.), consistent in direction with the main results.

## Discussion

### Main results

This study is the first to assess the role of national sick pay schemes for presenteeism propensity relying on a large European-wide data set. Findings underscore the relevance of sick pay generosity as a contextual factor shaping workers’ decisions to engage in presenteeism. After controlling for covariates, we found that generous sick pay was associated with an 8-percentage-point lower propensity for presenteeism, accounting for 12.4% of the variance in presenteeism propensity between countries. These results support the assumption that wage penalties associated with a lack of sick pay may encourage workers to engage in presenteeism [21, 35]. Our results align with previous research, notably Rostad *et al*.’s (2017) study of physicians in Sweden, Norway, and Italy, and Callison and Pesko’s (2022) and Vander Weerdt *et al*.’s (2023) investigation of paid sick leave mandates in the United States.

Generous sick pay appeared to be most important for workers who were older, reported greater financial difficulties, occupied lower occupational class positions, were employed in industry or public administration, or reported fewer health problems. In contrast, sick pay regulations seemed to be less relevant for managers, health care workers, and individuals with more frequent health events. These patterns are consistent with the expectation that sick pay generosity is particularly important for workers with limited economic resources. By contrast, managers and professionals may be more strongly influenced by performance pressure or career concerns than by immediate financial loss [6]. Sectoral differences further illustrate these dynamics: in health care, presenteeism often stems from workload pressure and understaffing [7], whereas in public administration or industry, where working conditions tend to be more stable, financial incentives appear more salient. Likewise, workers reporting a higher number of health events may be dealing with more serious or chronic conditions, where financial considerations play a lesser role.

From a policy perspective, these findings highlight the importance of income protection during sickness as a potential lever for reducing presenteeism. Strengthening sick pay schemes, particularly for low-income workers, may not only support better recovery but also help to prevent contagion in workplaces, especially during future pandemics [36].

### Strengths and limitations

Despite several strengths of the study, such as the large number of countries compared and the direct measure of presenteeism, the study also has limitations. The data were collected a decade ago, and the current context may have shifted, particularly in light of the COVID-19 pandemic, which may have altered workplace attitudes toward sickness absence and presenteeism. In addition, several countries extended sick pay or sickness benefit rules during the pandemic, although most of these adjustments were temporary [37, 38]. Based on our comparison with current regulations, statutory sick pay provisions appear to have remained relatively stable in most European countries since 2015. Notable exceptions are Ireland and the UK, which introduced or substantially extended statutory sick pay in 2023 and 2026, respectively [39]. In addition, the cross-sectional design of the study limits the ability to draw causal inferences. While we controlled for a wide range of observed characteristics, unobserved factors, such as individual traits, national work cultures, annual leave days or attitudes toward sickness absence, could still influence the results. Future research should employ longitudinal or quasi-experimental designs, as such data becomes available, which would enable more advanced methodological approaches.

## Conclusion

The findings of this study support the assumption that generous sick pay policies may discourage workers from engaging in presenteeism. Policy debates concerning the introduction or extension of waiting periods or reductions in wage replacement should consider that such measures may reduce sickness absenteeism at the cost of increased presenteeism.

## Supplementary Material

ckag093_Supplementary_Data

## Data Availability

The data underlying this article are available in the UK Data Service repository, at https://doi.org/10.5255/UKDA-SN-8098-5. Additional country-level indicators were obtained from the Sick Pay Database (https://doi.org/10.7910/DVN/ORQ66N), Eurostat (https://ec.europa.eu/eurostat/data/database), and the OECD Data Portal (https://data.oecd.org). Key points Presenteeism propensity varies considerably across European countries, with 9.5% of its variance attributable to the national context. More generous sick pay schemes are associated with significantly lower levels of presenteeism propensity, explaining 12.4% of the between-country variation. Workers in older age groups, low-income jobs, routine occupations, and industry or public administration are particularly sensitive to sick pay generosity. Generous sick pay regulations may help reduce financial pressures that drive workers to attend work while ill. Policy reforms that reduce wage replacement or introduce waiting periods should consider their potential to increase presenteeism and related health risks.
