# Supplementary material for: Variation in presenteeism by generosity of statutory sick pay: a multilevel analysis in 35 European countries
Source: Eur J Public Health. 2026 Jun 12;36(4):ckag093. doi: 10.1093/eurpub/ckag093 (PMC13262657; doi:10.1093/eurpub/ckag093)
Supplement: ckag093_Supplementary_Data [file ckag093_supplementary_data.zip › ejph-2025-11-om-0995-File011.docx]

Table S6 Multi-level generalised linear regression models for presenteeism propensity and their relation to alternative indicators for sick pay generosity

|  |  |  | **Model 5** | | **Model 6** | | **Model 7** | | **Model 8** | |
| --- | --- | --- | --- | --- | --- | --- | --- | --- | --- | --- |
|  |  |  | **AME** | **(SE)** | **AME** | **(SE)** | **AME** | **(SE)** | **AME** | **(SE)** |
| **Level 1 variables** | | | ✓ | | ✓ | | ✓ | | ✓ | |
| **Level 2 variables** | | | ✓ | | ✓ | | ✓ | | ✓ | |
|  | **Sick pay from day one** | |  |  |  |  |  |  |  |  |
|  |  | No | Ref. |  |  |  |  |  |  |  |
|  |  | Yes | -0.05 | (0.04) |  |  |  |  |  |  |
|  | **Wage replacement ≥ 80% at week 2** | |  |  |  |  |  |  |  |  |
|  |  | No |  |  | Ref. |  |  |  |  |  |
|  |  | Yes |  |  | -0.04 | (0.04) |  |  |  |  |
|  | **Sick pay independent of job tenure** | |  |  |  |  |  |  |  |  |
|  |  | No |  |  |  |  | Ref. |  |  |  |
|  |  | Yes |  |  |  |  | -0.09* | (0.04) |  |  |
|  | **Sick pay sum score (0-3)** | |  |  |  |  |  |  |  |  |
|  |  | 0 (most restrictive) |  |  |  |  |  |  | Ref. |  |
|  |  | 1 |  |  |  |  |  |  | -0.11 | (0.06) |
|  |  | 2 |  |  |  |  |  |  | -0.07 | (0.04) |
|  |  | 3 (most generous) |  |  |  |  |  |  | -0.14*** | (0.04) |
|  |  |  |  |  |  |  |  |  |  |  |
| **Intercept** | | | 0.59*** | 0.0 | 0.59*** | 0.0 | 0.59*** | 0.0 | 0.58*** | 0.0 |
| **Variance component** | | |  |  |  |  |  |  |  |  |
|  | **Level 1 (Individuals)** | | 2.700 | | 2.700 | | 2.700 | | 2.700 | |
|  | **Level 2 (Countries)** | | 0.188 | | 0.191 | | 0.170 | | 0.159 | |
|  |  | Intraclass correlation |  |  |  |  |  |  |  |  |
|  | **Variance reduction** | |  |  |  |  |  |  |  |  |
|  |  | Level 1 | 0.0% | | 0.0% | | 0.0% | | 0.0% | |
|  |  | Level 2 | -5.4% | | -3.7% | | -14.5% | | -19.8% | |
| **Model information** | | |  |  |  |  |  |  |  |  |
|  | **N (Individuals)** | | 19,657 | | 19,657 | | 19,657 | | 19,657 | |
|  | **N (Countries)** | | 35 | | 35 | | 35 | | 35 | |

Variance reduction in comparison to Model 2 in Table 2 or Table S4.

AME = Average marginal effect

SE = Standard error.

* p < 0.05, ** p < 0.01, *** p < 0.001.
