# Supplementary material for: Variation in presenteeism by generosity of statutory sick pay: a multilevel analysis in 35 European countries
Source: Eur J Public Health. 2026 Jun 12;36(4):ckag093. doi: 10.1093/eurpub/ckag093 (PMC13262657; doi:10.1093/eurpub/ckag093)
Supplement: ckag093_Supplementary_Data [file ckag093_supplementary_data.zip › ejph-2025-11-om-0995-File012.docx]

Table S7 Robustness analysis: Multilevel generalized linear regression models predicting absenteeism and presenteeism days

|  |  |  | **Model 9: Absenteeism days** | | **Model 10: Presenteeism days** | |
| --- | --- | --- | --- | --- | --- | --- |
|  |  |  | **AME** | **(SE)** | **AME** | **(SE)** |
| **Level 1 variables** | | | ✓ | | ✓ | |
| **Level 2 variables** | | |  |  |  |  |
|  | **Unemployment rate (Std.)** | | -0.73*** | (0.17) | 0.29 | (0.17) |
|  | **GDP per capita (Std.)** | | -0.64*** | (0.16) | 0.44** | (0.16) |
|  | **Population density (Std.)** | | -0.19 | (0.16) | 0.24 | (0.16) |
|  | **Generous sick pay** | |  |  |  |  |
|  |  | No | Ref. |  | Ref. |  |
|  |  | Yes | 0.58* | (0.29) | -0.49 | (0.28) |
|  |  |  |  |  |  |  |
| **Intercept** | | | 5.08*** | (0.16) | 3.20*** | (0.14) |
| **Model information** | | |  |  |  |  |
|  | **N (Individuals)** | | 19,657 | | 19,657 | |
|  | **N (Countries)** | | 35 | | 35 | |

Models were estimated using a negative binomial link function to account for the count nature of the dependent variables. SE = Standard error. * p < 0.05, ** p < 0.01, *** p < 0.001.
