# Supplementary material for: Variation in presenteeism by generosity of statutory sick pay: a multilevel analysis in 35 European countries
Source: Eur J Public Health. 2026 Jun 12;36(4):ckag093. doi: 10.1093/eurpub/ckag093 (PMC13262657; doi:10.1093/eurpub/ckag093)
Supplement: ckag093_Supplementary_Data [file ckag093_supplementary_data.zip › ejph-2025-11-om-0995-File006.docx]

Table S1 Frequencies of missing values before imputation

| **Variable** | **Complete (n)** | **Missing (n)** | **Missing (%)** | **Values** | **Min** | **Max** |
| --- | --- | --- | --- | --- | --- | --- |
| Age | 33,437 | 0 | 0.0 | 48 | 18 | 65 |
| Sex | 33,431 | 6 | 0.0 | 2 | 0 | 1 |
| Education | 33,333 | 104 | 0.3 | 3 | 1 | 3 |
| Household composition | 33,316 | 121 | 0.4 | 5 | 1 | 5 |
| Difficulty making ends meet | 33,185 | 252 | 0.8 | 3 | 1 | 3 |
| ESeC | 33,299 | 138 | 0.4 | 6 | 1 | 6 |
| NACE | 33,285 | 152 | 0.5 | 10 | 1 | 10 |
| Contract | 33,362 | 75 | 0.2 | 3 | 1 | 3 |
| Foreign born | 33,265 | 172 | 0.5 | 2 | 0 | 1 |
| Job tenure | 33,037 | 400 | 1.2 | 50 | 0 | 50 |
| Working hours | 32,828 | 609 | 1.9 | 75 | 1 | 80 |
| Company size | 32,738 | 699 | 2.1 | 3 | 1 | 3 |
| Union or works council representation | 31,984 | 1,453 | 4.5 | 2 | 0 | 1 |
| WHO-5 well-being index | 33,270 | 167 | 0.5 | 26 | 0 | 25 |
| Self-rated health | 33,404 | 33 | 0.1 | 5 | 1 | 5 |
| Long-standing illness (> 6 months) | 33,280 | 157 | 0.5 | 2 | 0 | 1 |
| Number of physical health problems | 33,221 | 216 | 0.7 | 8 | 0 | 7 |
| Days of sickness absence (last 12 months) | 29,999 | 3,438 | 11.5 | 114 | 0 | 365 |
| Days of sickness presence (last 12 months) | 32,069 | 1,368 | 4.3 | 79 | 0 | 365 |
| **Auxiliary variables** |  |  |  |  |  |  |
| Country | 33,437 | 0 | 0.0 | 35 | 1 | 35 |
| Region urbanisation | 33,437 | 0 | 0.0 | 3 | 1 | 3 |
| Multiple job holding | 33,350 | 87 | 0.3 | 2 | 0 | 1 |
| Occupational safety and health measures | 33,002 | 435 | 1.3 | 2 | 0 | 1 |
| Occupational safety and health risks at work | 32,895 | 542 | 1.6 | 2 | 0 | 1 |
| Ergonomic work demands | 33,226 | 211 | 0.6 | 5 | 0 | 4 |
| Environmental work demands | 33,041 | 396 | 1.2 | 10 | 0 | 9 |
| Working time demands | 31,507 | 1,930 | 6.1 | 7 | 0 | 6 |
| Psychological work demands | 32,063 | 1,374 | 4.3 | 11 | 0 | 10 |
| Decision latitude | 32,610 | 827 | 2.5 | 7 | 0 | 6 |
| Social support at work | 32,496 | 941 | 2.9 | 7 | 0 | 6 |
| Job rewards | 31,452 | 1,985 | 6.3 | 5 | 0 | 4 |

Descriptive statistics refer to the full sample of employed respondents before applying sample selection criteria. Auxiliary variables were included in the imputation models to enhance the accuracy of estimations. Continuous variables were imputed using predictive mean matching regression, binary variables by logistic regression, ordinal variables by ordinal logistic regression, and nominal categorical variables by multinomial regression.
