# Supplementary material for: Variation in presenteeism by generosity of statutory sick pay: a multilevel analysis in 35 European countries
Source: Eur J Public Health. 2026 Jun 12;36(4):ckag093. doi: 10.1093/eurpub/ckag093 (PMC13262657; doi:10.1093/eurpub/ckag093)
Supplement: ckag093_Supplementary_Data [file ckag093_supplementary_data.zip › ejph-2025-11-om-0995-File007.docx]

Table S2 Descriptive statistics on variables of interest before and after imputation

|  |  |  | **Before imputation** | | **After imputation** | |
| --- | --- | --- | --- | --- | --- | --- |
| **Variables** | | **Category or (range)** | **N (Col%)** | **Mean (SD)** | **N (Col%)** | **Mean (SD)** |
| **Socio-demographic factors** | |  |  |  |  |  |
|  | Age | (18-65) |  | 42.0 (11.5) |  | 42.0 (11.5) |
|  | Sex | Male | 16,132 (48.3) |  | 16,136 (48.3) |  |
|  |  | Female | 17,299 (51.7) |  | 17,301 (51.7) |  |
|  | Type of household | Single, no children | 5,066 (15.2) |  | 5,091 (15.2) |  |
|  |  | Couple, no children | 11,624 (34.9) |  | 11,667 (34.9) |  |
|  |  | Couple with children | 10,016 (30.1) |  | 10,053 (30.1) |  |
|  |  | Single with children | 1,531 (4.6) |  | 1,536 (4.6) |  |
|  |  | Others | 5,079 (15.2) |  | 5,090 (15.2) |  |
|  | Foreign born | No | 31,684 (95.2) |  | 31,837 (95.2) |  |
|  |  | Yes | 1,581 (4.8) |  | 1,600 (4.8) |  |
|  | Education (ISCED 2011) | Primary and lower secondary | 5,172 (15.5) |  | 5,195 (15.5) |  |
|  |  | Upper secondary | 16,535 (49.6) |  | 16,594 (49.6) |  |
|  |  | Tertiary | 11,626 (34.9) |  | 11,648 (34.8) |  |
|  | Difficulty making ends meet | Easily | 19,175 (57.8) |  | 19,302 (57.7) |  |
|  |  | Some difficulties | 12,447 (37.5) |  | 12,562 (37.6) |  |
|  |  | Great difficulties | 1,563 (4.7) |  | 1,573 (4.7) |  |
| **Job characteristics** | |  |  |  |  |  |
|  | Occupational class (ESeC) | Higher managers and professionals | 3,939 (11.8) |  | 3,957 (11.8) |  |
|  |  | Lower managers and professionals | 8,200 (24.6) |  | 8,219 (24.6) |  |
|  |  | Lower supervisors and technicians | 4,570 (13.7) |  | 4,581 (13.7) |  |
|  |  | Lower sales and service | 7,289 (21.9) |  | 7,309 (21.9) |  |
|  |  | Lower technical | 4,989 (15.0) |  | 5,031 (15.0) |  |
|  |  | Routine | 4,312 (12.9) |  | 4,340 (13.0) |  |
|  | Working sector (NACE) | Agriculture | 608 (1.8) |  | 612 (1.8) |  |
|  |  | Industry | 5,678 (17.1) |  | 5,706 (17.1) |  |
|  |  | Construction | 2,072 (6.2) |  | 2,091 (6.3) |  |
|  |  | Transport | 2,007 (6.0) |  | 2,014 (6.0) |  |
|  |  | Commerce and hospitality | 6,581 (19.8) |  | 6,613 (19.8) |  |
|  |  | Financial services | 1,200 (3.6) |  | 1,204 (3.6) |  |
|  |  | Public administration | 2,277 (6.8) |  | 2,285 (6.8) |  |
|  |  | Education | 3,340 (10.0) |  | 3,350 (10.0) |  |
|  |  | Health | 3,750 (11.3) |  | 3,759 (11.2) |  |
|  |  | Other services | 5,772 (17.3) |  | 5,803 (17.4) |  |
|  | Type of working contract | Permanent | 26,315 (78.9) |  | 26,362 (78.8) |  |
|  |  | Temporary | 4,212 (12.6) |  | 4,228 (12.6) |  |
|  |  | Other | 2,835 (8.5) |  | 2,847 (8.5) |  |
|  | Weekly working hours | (1-80) |  | 37.8 (10.8) |  | 37.9 (10.8) |
|  | Job tenure (years) | (0-50) |  | 9.6 (9.5) |  | 9.6 (9.5) |
|  | Company size | < 10 | 7,799 (23.8) |  | 8,018 (24.0) |  |
|  |  | 10-249 | 14,301 (43.7) |  | 14,615 (43.7) |  |
|  |  | 250+ | 10,638 (32.5) |  | 10,804 (32.3) |  |
|  | Union representation | No | 16,503 (51.6) |  | 17,436 (52.1) |  |
|  |  | Yes | 15,481 (48.4) |  | 16,001 (47.9) |  |
| **Health conditions** | |  |  |  |  |  |
|  | Self-rated health | (1=very bad to 5=very good) |  | 4.0 (0.7) |  | 4.0 (0.7) |
|  | Physical health problems | (0-7) |  | 1.8 (1.6) |  | 1.8 (1.6) |
|  | Long-standing illness | No | 27,614 (83.0) |  | 27,734 (82.9) |  |
|  |  | Yes | 5,666 (17.0) |  | 5,703 (17.1) |  |
|  | WHO-5 well-being index | (0=worst to 25=best) |  | 17.1 (5.0) |  | 17.1 (5.0) |
|  | Days of sickness absence | (0-365) |  | 6.1 (16.8) |  | 5.9 (16.5) |
|  | Days of sickness presence | (0-365) |  | 3.1 (11.2) |  | 3.2 (11.4) |
