# Supplementary material for: Variation in presenteeism by generosity of statutory sick pay: a multilevel analysis in 35 European countries
Source: Eur J Public Health. 2026 Jun 12;36(4):ckag093. doi: 10.1093/eurpub/ckag093 (PMC13262657; doi:10.1093/eurpub/ckag093)
Supplement: ckag093_Supplementary_Data [file ckag093_supplementary_data.zip › ejph-2025-11-om-0995-File008.docx]

Table S3 Presenteeism and sick pay regulations in 35 European countries (EWCS 2015)

| **Country** |  | **Presenteeism  propensity** | | **Sick pay from day one (No waiting period)** | **Wage replacement ≥ 80% at week 2** | **Generous  sick pay^a^** |
| --- | --- | --- | --- | --- | --- | --- |
|  | **N** | **Mean** | **(SD)** |  |  |  |
| Spain | 1,378 | 0.60 | (0.44) | No | No | No |
| France | 916 | 0.60 | (0.41) | No | Yes | No |
| United Kingdom | 917 | 0.55 | (0.40) | No | No | No |
| Serbia | 365 | 0.55 | (0.45) | Yes | No | No |
| North Macedonia | 272 | 0.51 | (0.46) | Yes | Yes | Yes |
| Luxembourg | 648 | 0.50 | (0.40) | Yes | Yes | Yes |
| Slovenia | 800 | 0.50 | (0.41) | Yes | Yes | Yes |
| Greece | 308 | 0.49 | (0.43) | Yes | No | No |
| Montenegro | 260 | 0.49 | (0.45) | Yes | No | No |
| Estonia | 442 | 0.48 | (0.43) | No | No | No |
| Ireland | 501 | 0.46 | (0.40) | No | No | No |
| Cyprus | 475 | 0.45 | (0.41) | No | No | No |
| Sweden | 666 | 0.45 | (0.38) | No | Yes | No |
| Denmark | 632 | 0.45 | (0.36) | Yes | Yes | Yes |
| Croatia | 439 | 0.44 | (0.43) | Yes | No | No |
| Malta | 707 | 0.44 | (0.38) | Yes | Yes | Yes |
| Norway | 613 | 0.43 | (0.39) | Yes | Yes | Yes |
| Belgium | 1,475 | 0.43 | (0.40) | Yes | No | No |
| Netherlands | 503 | 0.41 | (0.39) | Yes | No | No |
| Portugal | 210 | 0.40 | (0.46) | No | No | No |
| Hungary | 369 | 0.38 | (0.45) | Yes | No | No |
| Albania | 255 | 0.36 | (0.43) | Yes | Yes | Yes |
| Finland | 575 | 0.35 | (0.37) | Yes | Yes | Yes |
| Latvia | 335 | 0.34 | (0.43) | No | Yes | No |
| Slovakia | 435 | 0.31 | (0.38) | Yes | No | No |
| Austria | 498 | 0.31 | (0.38) | Yes | Yes | Yes |
| Poland | 416 | 0.28 | (0.40) | Yes | Yes | Yes |
| Bulgaria | 343 | 0.27 | (0.41) | Yes | Yes | Yes |
| Lithuania | 421 | 0.27 | (0.40) | Yes | Yes | Yes |
| Czech Republic | 448 | 0.27 | (0.39) | No | No | No |
| Romania | 253 | 0.26 | (0.40) | Yes | Yes | Yes |
| Turkey | 704 | 0.26 | (0.38) | No | No | No |
| Switzerland | 426 | 0.25 | (0.35) | Yes | Yes | Yes |
| Germany | 1,082 | 0.21 | (0.33) | Yes | Yes | Yes |
| Italy | 570 | 0.18 | (0.34) | No | No | No |
| **Total (Individuals)** | **19,657** | **0.41** | **(0.42)** | **0.62^b^** | **0.50^b^** | **0.40^b^** |
| **Total (Countries)** | **35** | **0.40** | **(0.03)** | **0.66^b^** | **0.51^b^** | **0.43^b^** |

^a^ Generous sick pay was defined as compensation from the first day of sickness, with wage replacement at 80% for at least two weeks. ^b^ Proportion of the 'Yes' category.
