# Supplementary material for: Variation in presenteeism by generosity of statutory sick pay: a multilevel analysis in 35 European countries
Source: Eur J Public Health. 2026 Jun 12;36(4):ckag093. doi: 10.1093/eurpub/ckag093 (PMC13262657; doi:10.1093/eurpub/ckag093)
Supplement: ckag093_Supplementary_Data [file ckag093_supplementary_data.zip › ejph-2025-11-om-0995-File009.docx]

Table S4 Multi-level generalised linear regression models for presenteeism propensity

|  |  |  | **Model 0** | | **Model 1** | | **Model 2** | | **Model 3** | |
| --- | --- | --- | --- | --- | --- | --- | --- | --- | --- | --- |
|  |  |  | **AME** | **(SE)** | **AME** | **(SE)** | **AME** | **(SE)** | **AME** | **(SE)** |
| **Level 1 variables** | | |  |  |  |  |  |  |  |  |
|  | **Age (Std.)** | |  |  | -0.03*** | (0.00) | -0.03*** | (0.00) | -0.03*** | (0.00) |
|  | **Sex** | |  |  |  |  |  |  |  |  |
|  |  | Male |  |  | Ref. |  | Ref. |  | Ref. |  |
|  |  | Female |  |  | 0.03*** | (0.01) | 0.03*** | (0.01) | 0.03*** | (0.01) |
|  | **Type of household** | |  |  |  |  |  |  |  |  |
|  |  | Single, no children |  |  | Ref. |  | Ref. |  | Ref. |  |
|  |  | Couple, no children |  |  | 0.00 | (0.01) | 0.00 | (0.01) | 0.00 | (0.01) |
|  |  | Couple with children |  |  | 0.02 | (0.01) | 0.02 | (0.01) | 0.02 | (0.01) |
|  |  | Single with children |  |  | 0.01 | (0.01) | 0.01 | (0.01) | 0.01 | (0.01) |
|  |  | Others |  |  | 0.00 | (0.01) | 0.00 | (0.01) | 0.00 | (0.01) |
|  | **Foreign born** | |  |  |  |  |  |  |  |  |
|  |  | No |  |  | Ref. |  | Ref. |  | Ref. |  |
|  |  | Yes |  |  | 0.02 | (0.02) | 0.02 | (0.02) | 0.02 | (0.02) |
|  | **Education (ISCED 2011)** | |  |  |  |  |  |  |  |  |
|  |  | Primary and lower secondary |  |  | Ref. |  | Ref. |  | Ref. |  |
|  |  | Upper secondary |  |  | 0.00 | (0.01) | 0.00 | (0.01) | 0.00 | (0.01) |
|  |  | Tertiary |  |  | 0.02 | (0.01) | 0.02 | (0.01) | 0.02 | (0.01) |
|  | **Difficulty making ends meet** | |  |  |  |  |  |  |  |  |
|  |  | Easily |  |  | Ref. |  | Ref. |  | Ref. |  |
|  |  | Some difficulties |  |  | 0.02 | (0.01) | 0.02 | (0.01) | 0.02 | (0.01) |
|  |  | Great difficulties |  |  | 0.10*** | (0.02) | 0.10*** | (0.02) | 0.10*** | (0.02) |
|  | **Occupational class (ESeC)** | |  |  |  |  |  |  |  |  |
|  |  | Higher managers and professionals | |  | Ref. |  | Ref. |  | Ref. |  |
|  |  | Lower managers and professionals | |  | -0.04** | (0.01) | -0.04** | (0.01) | -0.04** | (0.01) |
|  |  | Lower supervisors and technicians | |  | -0.07*** | (0.01) | -0.07*** | (0.01) | -0.07*** | (0.01) |
|  |  | Lower sales and service |  |  | -0.07*** | (0.02) | -0.07*** | (0.02) | -0.07*** | (0.02) |
|  |  | Lower technical |  |  | -0.12*** | (0.02) | -0.12*** | (0.02) | -0.12*** | (0.02) |
|  |  | Routine |  |  | -0.11*** | (0.02) | -0.11*** | (0.02) | -0.11*** | (0.02) |
|  | **Working sector (NACE)** | |  |  |  |  |  |  |  |  |
|  |  | Agriculture |  |  | 0.03 | (0.03) | 0.03 | (0.03) | 0.03 | (0.03) |
|  |  | Industry |  |  | 0.00 | (0.02) | 0.00 | (0.02) | 0.00 | (0.02) |
|  |  | Construction |  |  | -0.01 | (0.02) | -0.01 | (0.02) | -0.01 | (0.02) |
|  |  | Transport |  |  | 0.00 | (0.02) | 0.00 | (0.02) | 0.00 | (0.02) |
|  |  | Commerce and hospitality |  |  | 0.01 | (0.01) | 0.01 | (0.01) | 0.01 | (0.01) |
|  |  | Financial services |  |  | 0.01 | (0.02) | 0.01 | (0.02) | 0.01 | (0.02) |
|  |  | Public administration |  |  | 0.02 | (0.02) | 0.02 | (0.02) | 0.02 | (0.02) |
|  |  | Education |  |  | 0.06*** | (0.01) | 0.06*** | (0.01) | 0.06*** | (0.01) |
|  |  | Health |  |  | Ref. |  | Ref. |  | Ref. |  |
|  |  | Other services |  |  | 0.01 | (0.01) | 0.01 | (0.01) | 0.01 | (0.01) |
|  | **Type of working contract** | |  |  |  |  |  |  |  |  |
|  |  | Permanent |  |  | Ref. |  | Ref. |  | Ref. |  |
|  |  | Temporary |  |  | 0.05*** | (0.01) | 0.05*** | (0.01) | 0.05*** | (0.01) |
|  |  | Other |  |  | 0.03 | (0.02) | 0.03 | (0.02) | 0.03 | (0.02) |
|  | **Weekly working hours (Std.)** | |  |  | 0.03*** | (0.01) | 0.03*** | (0.01) | 0.03*** | (0.01) |
|  | **Job tenure (Std.)** | |  |  | 0.00 | (0.01) | 0.00 | (0.01) | 0.00 | (0.01) |
|  | **Company size** | |  |  |  |  |  |  |  |  |
|  |  | < 10 |  |  | Ref. |  | Ref. |  | Ref. |  |
|  |  | 10-249 |  |  | 0.00 | (0.01) | 0.00 | (0.01) | 0.00 | (0.01) |
|  |  | 250+ |  |  | 0.03* | (0.01) | 0.03* | (0.01) | 0.03* | (0.01) |
|  | **Union or works council** | |  |  |  |  |  |  |  |  |
|  |  | No |  |  | Ref. |  | Ref. |  | Ref. |  |
|  |  | Yes |  |  | -0.02** | (0.01) | -0.02** | (0.01) | -0.02** | (0.01) |
|  | **Self-rated health (Std.)** | |  |  | 0.01 | (0.01) | 0.01 | (0.01) | 0.01 | (0.01) |
|  | **Physical health problems (Std.)** | |  |  | 0.07*** | (0.01) | 0.07*** | (0.01) | 0.07*** | (0.01) |
|  | **Long-standing illness** | |  |  |  |  |  |  |  |  |
|  |  | No |  |  | Ref. |  | Ref. |  | Ref. |  |
|  |  | Yes |  |  | 0.04** | (0.01) | 0.04** | (0.01) | 0.04** | (0.01) |
|  | **WHO-5 well-being index (Std.)** | |  |  | -0.04*** | (0.01) | -0.04*** | (0.01) | -0.04*** | (0.01) |
|  | **Health events (Std.)** | |  |  | 0.04*** | (0.01) | 0.04*** | (0.01) | 0.04*** | (0.01) |
|  |  |  |  |  |  |  |  |  |  |  |
| **Level 2 variables** | | |  |  |  |  |  |  |  |  |
|  | **Unemployment rate (Std.)** | |  |  |  |  | 0.05*** | (0.02) | 0.05*** | (0.01) |
|  | **GDP per capita (Std.)** | |  |  |  |  | 0.07*** | (0.02) | 0.08*** | (0.02) |
|  | **Population density (Std.)** | |  |  |  |  | 0.04*** | (0.01) | 0.04*** | (0.01) |
|  | **Generous sick pay** | |  |  |  |  |  |  |  |  |
|  |  | No |  |  |  |  |  |  | Ref. |  |
|  |  | Yes |  |  |  |  |  |  | -0.08* | (0.04) |
|  |  |  |  |  |  |  |  |  |  |  |
| **Intercept** | | | 0.57*** | (0.02) | 0.57*** | (0.02) | 0.59*** | (0.02) | 0.59*** | 0.0 |
| **Variance component** | | |  |  |  |  |  |  |  |  |
|  | **Level 1 (Individuals)** | | 3.290 | | 2.700 | | 2.700 | | 2.700 | |
|  | **Level 2 (Countries)** | | 0.345 | | 0.293 | | 0.199 | | 0.174 | |
|  |  | Intraclass correlation | 0.095 | |  |  |  |  |  |  |
|  | **Variance reduction** | |  |  |  |  |  |  |  |  |
|  |  | Level 1 |  |  | -17.9% | | 0.0% | | 0.0% | |
|  |  | Level 2 |  |  | -15.1% | | -32.2% | | -12.4% | |
| **Model information** | | |  |  |  |  |  |  |  |  |
|  | **N (Individuals)** | | 19,657 | | 19,657 | | 19,657 | | 19,657 | |
|  | **N (Countries)** | | 35 | | 35 | | 35 | | 35 | |

AME = average marginal effect (this represents the average change in the predicted fraction of days worked while sick, expressed in percentage points; for example, women had a 3–percentage‐point higher fraction of presenteeism days than men). SE=Standard Error. Continuous variables have been standardised (Std.). Variances have been rescaled following the McKelvey and Zavoina method (as described in Hox, 2010, pp. 133–139) to make them comparable across different logit models. ISCED=International Standard Classification of Education. ESeC = European Socio-economic Classification.

NACE = Statistical Classification of Economic Activities in the European Community.

* p < 0.05, ** p < 0.01, *** p < 0.001
