# Supplementary material for: Variation in presenteeism by generosity of statutory sick pay: a multilevel analysis in 35 European countries
Source: Eur J Public Health. 2026 Jun 12;36(4):ckag093. doi: 10.1093/eurpub/ckag093 (PMC13262657; doi:10.1093/eurpub/ckag093)
Supplement: ckag093_Supplementary_Data [file ckag093_supplementary_data.zip › ejph-2025-11-om-0995-File010.docx]

Table S5 Multi-level generalised linear regression models for presenteeism propensity and cross-level interaction between sick pay and individual-level factors

|  |  |  | **Model 4a** | | **Model 4b** | | **Model 4c** | | **Model 4d** | | **Model 4e** | | **Model 4f** | |
| --- | --- | --- | --- | --- | --- | --- | --- | --- | --- | --- | --- | --- | --- | --- |
|  |  |  | **Coef** | **(SE)** | **Coef** | **(SE)** | **Coef** | **(SE)** | **Coef** | **(SE)** | **Coef** | **(SE)** | **Coef** | **(SE)** |
| **Level 1 variables** | | |  |  |  |  |  |  |  |  |  |  |  |  |
|  | **Age group** | |  |  |  |  |  |  |  |  |  |  |  |  |
|  |  | 18-29 years | Ref. |  | Ref. |  | Ref. |  | Ref. |  | Ref. |  | Ref. |  |
|  |  | 30-44 years | -0.12* | (0.05) | -0.13* | (0.06) | -0.12* | (0.05) | -0.12* | (0.05) | -0.12* | (0.05) | -0.12* | (0.05) |
|  |  | 45-59 years | -0.25*** | (0.06) | -0.24*** | (0.06) | -0.25*** | (0.06) | -0.25*** | (0.06) | -0.25*** | (0.06) | -0.25*** | (0.06) |
|  |  | 60-65 years | -0.43*** | (0.09) | -0.36** | (0.12) | -0.43*** | (0.09) | -0.43*** | (0.09) | -0.43*** | (0.09) | -0.43*** | (0.09) |
|  | **Sex** | |  |  |  |  |  |  |  |  |  |  |  |  |
|  |  | Male | Ref. |  | Ref. |  | Ref. |  | Ref. |  | Ref. |  | Ref. |  |
|  |  | Female | 0.13* | (0.05) | 0.17*** | (0.04) | 0.17*** | (0.04) | 0.17*** | (0.04) | 0.17*** | (0.04) | 0.17*** | (0.04) |
|  | **Difficulty making ends meet** | | |  |  |  |  |  |  |  |  |  |  |  |
|  |  | Easily | Ref. |  | Ref. |  | Ref. |  | Ref. |  | Ref. |  | Ref. |  |
|  |  | Some difficulties | 0.06 | (0.04) | 0.06 | (0.04) | 0.05 | (0.06) | 0.06 | (0.04) | 0.06 | (0.04) | 0.06 | (0.04) |
|  |  | Great difficulties | 0.46*** | (0.09) | 0.46*** | (0.09) | 0.50*** | (0.12) | 0.46*** | (0.09) | 0.46*** | (0.09) | 0.46*** | (0.09) |
|  | **Occupational class (ESeC)** | | |  |  |  |  |  |  |  |  |  |  |  |
|  |  | Manager/Prof. | Ref. |  | Ref. |  | Ref. |  | Ref. |  | Ref. |  | Ref. |  |
|  |  | Intermediate | -0.27*** | (0.04) | -0.27*** | (0.04) | -0.27*** | (0.04) | -0.27*** | (0.04) | -0.23*** | (0.05) | -0.27*** | (0.04) |
|  |  | Routine | -0.26*** | (0.05) | -0.26*** | (0.05) | -0.27*** | (0.05) | -0.26*** | (0.05) | -0.22*** | (0.06) | -0.26*** | (0.05) |
|  | **Working sector (NACE)** | | |  |  |  |  |  |  |  |  |  |  |  |
|  |  | Manual/Prod. | Ref. |  | Ref. |  | Ref. |  | Ref. |  | Ref. |  | Ref. |  |
|  |  | Services | 0.11* | (0.05) | 0.11* | (0.05) | 0.11* | (0.05) | 0.05 | (0.06) | 0.11* | (0.05) | 0.11* | (0.05) |
|  |  | Pub. admin. | 0.12* | (0.06) | 0.12* | (0.06) | 0.12* | (0.06) | 0.12 | (0.07) | 0.12* | (0.06) | 0.12* | (0.05) |
|  |  | Education | 0.27*** | (0.07) | 0.27*** | (0.07) | 0.27*** | (0.07) | 0.20* | (0.08) | 0.27*** | (0.07) | 0.27*** | (0.07) |
|  |  | Health | 0.01 | (0.07) | 0.01 | (0.07) | 0.02 | (0.07) | -0.13 | (0.08) | 0.02 | (0.07) | 0.02 | (0.07) |
|  | **Health events** | |  |  |  |  |  |  |  |  |  |  |  |  |
|  |  | 1-7 days | Ref. |  | Ref. |  | Ref. |  | Ref. |  | Ref. |  | Ref. |  |
|  |  | 8-14 days | 0.49*** | (0.08) | 0.49*** | (0.08) | 0.49*** | (0.08) | 0.49*** | (0.08) | 0.49*** | (0.08) | 0.44*** | (0.10) |
|  |  | 15-30 days | 0.45*** | (0.09) | 0.45*** | (0.09) | 0.45*** | (0.09) | 0.45*** | (0.09) | 0.45*** | (0.09) | 0.40*** | (0.11) |
|  |  | >30 days | 0.56*** | (0.13) | 0.56*** | (0.14) | 0.56*** | (0.13) | 0.56*** | (0.14) | 0.56*** | (0.13) | 0.39* | (0.15) |
| **Level 2 variables** | | |  |  |  |  |  |  |  |  |  |  |  |  |
|  | **Generous sick pay** | |  |  |  |  |  |  |  |  |  |  |  |  |
|  |  | No | Ref. |  | Ref. |  | Ref. |  | Ref. |  | Ref. |  | Ref. |  |
|  |  | Yes | -0.44* | (0.18) | -0.36* | (0.17) | -0.39* | (0.18) | -0.50** | (0.19) | -0.32 | (0.16) | -0.45* | (0.18) |
| **Interaction** | | |  |  |  |  |  |  |  |  |  |  |  |  |
|  | **Generous sick pay # Sex** | | |  |  |  |  |  |  |  |  |  |  |  |
|  |  | Yes # Female | 0.11 | (0.07) |  |  |  |  |  |  |  |  |  |  |
|  | **Generous sick pay # Age group** | | |  |  |  |  |  |  |  |  |  |  |  |
|  |  | Yes # 30-44 years | 0.01 | (0.11) |  |  |  |  |  |  |  |  |  |  |
|  |  | Yes # 45-59 years | -0.03 | (0.12) |  |  |  |  |  |  |  |  |  |  |
|  |  | Yes # 60-65 years | -0.16 | (0.18) |  |  |  |  |  |  |  |  |  |  |
|  | **Generous sick pay # Age group** | | |  |  |  |  |  |  |  |  |  |  |  |
|  |  | Yes # 30-44 years |  |  | 0.01 | (0.11) |  |  |  |  |  |  |  |  |
|  |  | Yes # 45-59 years |  |  | -0.03 | (0.12) |  |  |  |  |  |  |  |  |
|  |  | Yes # 60-65 years |  |  | -0.16 | (0.18) |  |  |  |  |  |  |  |  |
|  | **Generous sick pay # Ends meet** | | |  |  |  |  |  |  |  |  |  |  |  |
|  |  | Yes # Some difficulties | |  |  |  | 0.03 | (0.09) |  |  |  |  |  |  |
|  |  | Yes # Great difficulties | |  |  |  | -0.13 | (0.18) |  |  |  |  |  |  |
|  | **Generous sick pay # Working sector** | | | |  |  |  |  |  |  |  |  |  |  |
|  |  | Yes # Services |  |  |  |  |  |  | 0.16* | (0.07) |  |  |  |  |
|  |  | Yes # Pub. admin. |  |  |  |  |  |  | -0.04 | (0.11) |  |  |  |  |
|  |  | Yes # Education |  |  |  |  |  |  | 0.16 | (0.12) |  |  |  |  |
|  |  | Yes # Health |  |  |  |  |  |  | 0.34** | (0.12) |  |  |  |  |
|  | **Generous sick pay # Occupational class** | | | |  |  |  |  |  |  |  |  |  |  |
|  |  | Yes # Intermediate |  |  |  |  |  |  |  |  | -0.09 | (0.08) |  |  |
|  |  | Yes # Routine |  |  |  |  |  |  |  |  | -0.11 | (0.10) |  |  |
|  | **Generous sick pay # Health events** | | |  |  |  |  |  |  |  |  |  |  |  |
|  |  | Yes # 8-14 days |  |  |  |  |  |  |  |  |  |  | 0.11 | (0.16) |
|  |  | Yes # 15-30 days |  |  |  |  |  |  |  |  |  |  | 0.12 | (0.17) |
|  |  | Yes # >30 days |  |  |  |  |  |  |  |  |  |  | 0.40 | (0.25) |
| **Model information** | | |  |  |  |  |  |  |  |  |  |  |  |  |
|  | **N (Individuals)** | | 19,657 | | 19,657 | | 19,657 | | 19,657 | | 19,657 | | 19,657 | |
|  | **N (Countries)** | | 35 | | 35 | | 35 | | 35 | | 35 | | 35 | |

Each model includes the full set of covariates from the Model 3 shown in Table S3. Coef = Log-odds. SE = Standard error. * p < 0.05, ** p < 0.01, *** p < 0.001.
